# *GATA3* as an Adjunct Prognostic Factor in Breast Cancer Patients with Less Aggressive Disease: A Study with a Review of the Literature

**DOI:** 10.3390/diagnostics11040604

**Published:** 2021-03-28

**Authors:** Patrizia Querzoli, Massimo Pedriali, Rosa Rinaldi, Paola Secchiero, Paolo Giorgi Rossi, Elisabetta Kuhn

**Affiliations:** 1 Section of Anatomic Pathology, Department of Morphology, Surgery and Experimental Medicine, University of Ferrara, 44124 Ferrara, Italy; patrizia.querzoli@unife.it (P.Q.); mpedriali@gmail.com (M.P.); 2 Section of Anatomic Pathology, ASST Mantova, Ospedale Carlo Poma, 46100 Mantova, Italy; rosa.rinaldi@asst-mantova.it; 3 Surgery and Experimental Medicine and Interdepartmental Center of Technology of Advanced Therapies (LTTA), Department of Morphology, University of Ferrara, 44121 Ferrara, Italy; paola.secchiero@unife.it; 4 Epidemiology Unit, Azienda Unità Sanitaria Locale-IRCCS di Reggio Emilia, 42122 Reggio Emilia, Italy; paolo.giorgirossi@ausl.re.it; 5 Division of Pathology, Fondazione IRCCS Ca’ Granda, Ospedale Maggiore Policlinico, 20122 Milano, Italy; 6 Department of Biomedical, Surgical, and Dental Sciences, University of Milan, 20122 Milano, Italy

**Keywords:** breast cancer, breast, *GATA3*, prognosis, prognostic factor, biomarker

## Abstract

Background: GATA binding protein 3 (*GATA3*) expression is positively correlated with estrogen receptor (ER) expression, but its prognostic value as an independent factor remains unclear. Thus, we undertook the current study to evaluate the expression of *GATA3* and its prognostic value in a large series of breast carcinomas (BCs) with long-term follow-up. Methods: A total of 702 consecutive primary invasive BCs resected between 1989 and 1993 in our institution were arranged in tissue microarrays, immunostained for ER, progesterone receptor (PR), ki-67, HER2, p53, and *GATA3*, and scored. Clinico-pathological data were retrospectively collected. Results: *GATA3* was evaluable in 608 (87%) of the 702 cases; it was positive in 413 (68%) cases and negative in 195 (32%) cases. *GATA3* positivity was significantly associated with lower grade (*p* < 0.0001), size (*p* = 0.0463), stage (*p* = 0.0049), ER+ (*p* < 0.0001), PR+ (*p* < 0.0001), HER2− (*p* = 0.0175), and p53 wild-type pattern (*p* < 0.0001). The median follow-up was 183 months, *GATA3* positivity was associated with better overall survival (HR 0.70, *p* = 0.001), and its prognostic value was retained in a multivariate analysis. The association with better overall survival was stronger in patients with grade 1–2, pT1–2, pN0, stage I–II, ER+, PR+, ki-67 < 20%, HER2−, a wild-type p53 immunohistochemical pattern, and in luminal B BC. Conclusions: Our findings indicate that *GATA3* is a positive prognostic marker in BC patients, especially in patients with biologically less aggressive BC. Incorporating *GATA3* immunohistochemistry into routine practice could help further stratify BC patients for their risk.

## 1. Introduction

Medical oncologists treating breast cancer (BC) patients rely on well-known prognostic and predictive factors in order to provide the most appropriate and individualized clinical management. The main clinico-pathological features of BC with a recognized prognostic value include tumor size, axillary lymph node status, and histological grade, while hormone receptor and HER2 statuses effectively predict the response to hormonal or anti-HER2 target therapies, respectively [1,2,3]. More recently, multigene tests have been proposed to predict the prognosis and response to chemotherapy, and have been entered in several recommendations [4,5], but their high costs reduce the opportunity for routine use [4]. However, individual outcome differences cannot be fully explained only by single factors by these multigene tests. Therefore, additional and affordable prognostic factors are still needed.

GATA binding protein 3 (*GATA3*), a member of the GATA family, plays a pivotal role in the tissue determination of many organs, in particular T cells and the mammary gland [6,7,8]. Specifically, *GATA3* is critical for the luminal differentiation of breast epithelial cells and in the morphogenesis of the mammary gland [6,8]. Coherently, *GATA3* expression in human BCs is positively associated with estrogen receptor (ER) expression, and is required for cell cycle progression of ER-positive cell lines and coregulated with ER. On the other hand, it has been found that, in ER-negative cell lines, *GATA3* interacts with wild-type BRCA1, but it is incapable of binding to mutant BRCA1 [9]. This finding is important, since the disruption of the BRCA1/*GATA3* interaction could be the biochemical mechanism underlining the aggressive behavior of basal-like BC [9]. Moreover, *GATA3* lacks leads to a chemo-resistant and mesenchymal phenotype of BC [10,11]. Functionally, *GATA3* expression in BC cell lines and murine models reduces the tumor-initiating ability, the epithelial to mesenchymal transition, and the metastatic potential [12].

The importance of *GATA3* in breast biology is further emphasized by the fact that *GATA3* is one of the few recurrently mutated genes in BC, occurring in 12–16% of BCs across all different subtypes [13,14]. Interestingly, specific *GATA3* mutations have been identified in causing a congenital developmental disorder characterized by hypoparathyroidism, sensorineural deafness, and renal insufficiency (so-called HDR syndrome). Similarly to HDR syndrome mutations, most of the *GATA3* somatic mutations found in BC hit the C-terminal zinc finger region, revealing the key role this region has in the normal functionality of the protein [13,14].

Many studies have investigated *GATA3* as a prognostic marker in BC patients, with conflicting findings. On one hand, *GATA3* mutations and gene expression have been associated with better prognosis and improved survival [15,16,17]. On the other hand, *GATA3* expression closely parallels ER expression in BCs, so that it does not seem to be a prognostic factor independent of ER status [18]. Hence, whether or not *GATA3* carries independent prognostic information in BC patients remains an open question. As a consequence, we undertook the current study to evaluate the expression of *GATA3* by immunohistochemistry (IHC) in a large consecutive series of invasive BCs with long-term follow-up and to correlate that with clinico-pathological characteristics, biological markers, p53 status, and clinical outcome, in order to better clarify whether *GATA3* carries independent prognostic information.

## 2. Materials and Methods

### 2.1. Patients

Eligible patients were all female BC patients diagnosed and surgically treated from January 1989 to December 1993 at the surgical units of the S. Anna University Hospital of Ferrara or at other hospitals in the Ferrara province. Exclusion criteria were not having available tumor formalin-fixed and paraffin-embedded blocks, undergoing neoadjuvant treatment, and having systemic metastasis at diagnosis. A total of 702 consecutive primary invasive BCs were retrieved from the pathological files of our institution and included in this study.

All patients were treated according to our institution guidelines before the introduction of the current therapy based on BC molecular intrinsic subtypes. After the first-line treatment was completed, patients were re-examined twice a year for the first five years, then annually for the following years. Clinical baseline data, including patient age, tumor histology, pathologic stage, grading, and follow-up data (last follow-up time, date of death, and cause of death), were retrospectively collected from the pathology files and the Ferrara Cancer Registry. Tissue collection was conformed to the Institutional Research Board regulations of the University Hospitals of Ferrara. The protocol of this study was approved by the board of the Ministry of University and Research (identification and validation of new markers of metastasizing phenotype of breast cancer, prot. MM06095812_006, 2000).

### 2.2. Tissue Microarray Construction and Immunohistochemical Staining

Tissue microarrays (TMAs) were prepared as previously described [19]. The complete tumor series was included in a total of 31 TMA blocks. Consecutive 4-μm thick sections were mounted on silanized slides.

TMA sections were stained either manually or by the Ventana NexES automated immunostainer (Ventana Medical Systems/Roche, Tucson, AZ, USA) using the primary antibodies and conditions reported in Table S1 and the Supplementary Methods Section. ER, PR, HER2, ki67, p53, and *GATA3* IHC staining was reported as the proportion of positive cells. Moreover, a histological score was obtained for *GATA3* as the product of the percentage of positive cell nuclei (0–100%) and the four-tier intensity score (0, 1+, 2+, 3+). Therefore, the final histological score ranged from 0 to 300 for each core. The IHC scoring and positivity definition threshold are detailed in the Supplementary Methods Section and in Table S1.

### 2.3. Variables of Interest

All tumors were categorized according to the WHO classification and the eighth AJCC staging system, and graded according to the Elston–Ellis grading system [20,21]; single components of TNM staging are also reported. Histotype was categorized as no special type, lobular, or other special types (including tubular, mucinous, papillary, medullary, cribriform, apocrine, and micropapillary carcinoma). The molecular classification of BC was based on surrogate definitions by means of IHC markers for ER, PR, HER2, and ki-67, according to the criteria of the 2011 St. Gallen International Breast Cancer Conference [2].

### 2.4. Outcome

Survival time to death (for any cause) was used as the outcome variable. The date of death was extracted from the local cancer registry.

### 2.5. Statistical Analysis

For the association between *GATA3* positivity and categorical variables, the chi-squared test (or Fisher’s exact test, when appropriate) was used to estimate the *p*-value, i.e., the probability of obtaining the observed (or stronger) association under the null hypothesis. For associations between continuous and categorical variables, the Student’s *t*-test (or the Wilcoxon’s signed rank test, when appropriate) was used. The survival curves were estimated using the Kaplan–Meier method, and the log-rank test was used to measure the *p*-value. All of the markers, including ER, PR, HER2, Ki67, p53, and *GATA3*, were included, together with age, stage, and grade in a multivariate analysis using the Cox regression model. Hazard ratios (HRs) with 95% confidence intervals (CIs) were used to quantify the prognostic impact of variables. Variables with a *p*-value larger than 0.2 were excluded from the model using a backward variable selection strategy. Proportional hazard assumption was visually checked using the log-log plot and the predicted vs. observed curve comparison.

The prognostic value of *GATA3* in predicting OS, plotted as Kaplan–Meier survival curves, is also reported as stratified by the other main prognostic factors.

The *p*-values, as continuous variables, and 95% CIs are reported in order to show the probability that a difference or an association is observed for a random fluctuation under the null hypothesis, but no formal statistical test has been performed. All statistical analyses were conducted using Stata version 13.0 (StataCorp, College Station, TX, USA), and GraphPad Prism 5 software (GraphPad Software, Inc., San Diego, CA, USA) was used to plot and compare the data.

## 3. Results

### 3.1. Association between GATA3 Expression and Clinico-Pathological Features

Clinico-pathological and IHC characteristics of the 702 patients included in this study are summarized in Table 1 and detailed in the Supplementary Materials Section.

**Figure 1:**
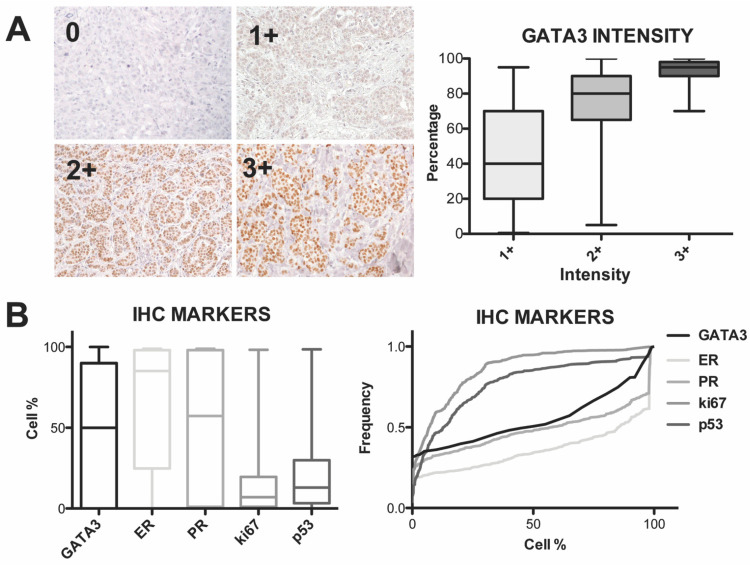
Representative pictures of immunostaining intensity scores of *GATA3* in BC tissues and box plot diagrams (top left and top right panels, respectively) illustrating the results of *GATA3* staining of 608 BC samples as positive cell percentage according to intensity scores. The differences between percent positive cells based on intensity were significant (*p*-value < 0.0001, Mann–Whitney U test, two-sided, right panel) (**A**)**.** Box plot (bottom left) and cumulative relative frequency charts (bottom right) showing the percentage distribution of the immunohistochemical markers, including estrogen receptors (ER), progesterone receptor (PR), Ki-67 proliferation index, p53 oncosuppressor gene, and *GATA3* (**B**).

Overall, *GATA3* was evaluable in 608 (87%) of the 702 BC cases, and was positive (≥1%) in 413 (68%) cases and negative (<1%) in 195 (32%) cases, with a *GATA3* median percentage of 50% (range 0–100%) and a median histological score of 60 (Figure 1A). The remaining 94 TMA cores (13.4%) were not evaluable for *GATA3* due to tissue loss, unrepresentative tissue, or non-specific staining.

**Figure 2:**
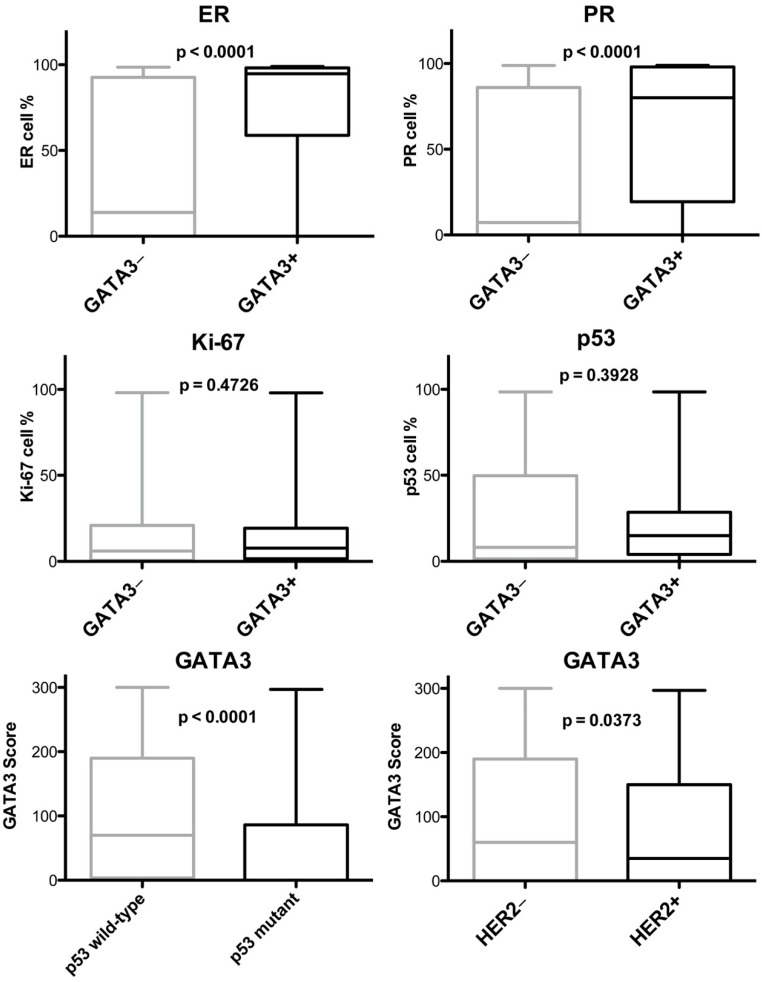
Box-plot charts showing the correlation between *GATA3* expression (i.e., − is < 1%; + is ≥ 1%) and the biological factors ER, PR, proliferative index Ki-67, and p53, and the box plot chart of the *GATA3* histological score according to p53 immunohistochemical pattern and HER2 status; *p*-values from the two-sided *t*-test comparisons are reported.

*GATA3* IHC positivity was associated with ER (*p* < 0.0001), PR (*p* < 0.0001), p53 wild-type pattern (*p* < 0.0001), and HER2 negative status (*p* = 0.0175) (Supplementary Table S2 and Figure 1B and Figure 2). A weak association was found between the *GATA3* and ki67 proliferation index (*p* = 0.3575).

**Figure 3:**
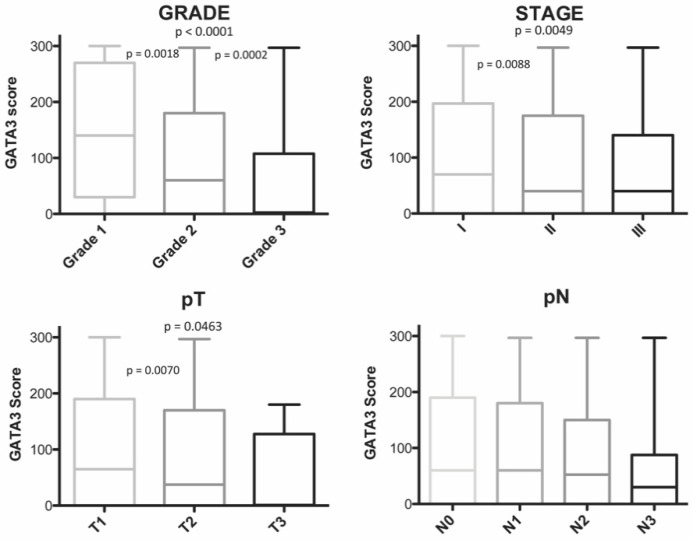
Box plot showing the correlation between *GATA3* score and histological grade, stage grouping, pathological primary tumor size (pT), and pathological lymph node involvement (pN). Significant *p*-values from two-sided *t*-test comparisons are reported.

*GATA3* positivity decreased with histological grade (*p* < 0.0001), stage grouping (*p* = 0.0049), and pT staging (*p* = 0.0463) (Figure 3). Therefore, *GATA3* expression was higher in BCs with less aggressive clinico-pathological characteristics, such as grade 1 and grade 2, smaller tumor size, and lower stage than in BCs with worse prognosis characteristics. In contrast, *GATA3* expression showed almost no association with age and pN staging (*p* = 0.3695 and *p* = 0.845).

### 3.2. Association between GATA3 Expression and Molecular Subtypes

Among the BCs with scored *GATA3*, 576 cases were subclassified in molecular subtypes according to IHC biological markers (Table S3 and Figure S1). *GATA3* positivity was higher in luminal A, luminal B-HER2–, and luminal B-HER2+ subtypes (chi-squared test, *p* < 0.0001).

### 3.3. GATA3 Expression as a Predictor of Prognosis

**Figure 4:**
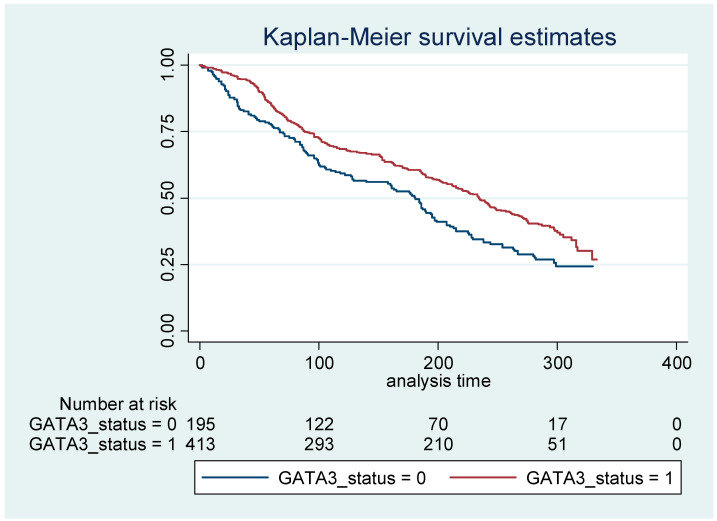
Kaplan–Meier survival curve analysis of *GATA3* in breast carcinomas. The age-adjusted hazard ratio for death was 0.70 (95% confidence interval 0.56–0.86).

In our patients with a median follow-up of 183 months, *GATA3* IHC positivity was associated with better overall survival (OS, median 234 vs. 181 months for *GATA3*+ vs. *GATA3*–). Specifically, after adjusting for age, the overall hazard ratio for death was 0.70 (95% CI: 0.56 to 0.86, *p* = 0.001) for BC patients with positive *GATA3* when compared with negative *GATA3* (Figure 4 and Table S4).

**Figure 5:**
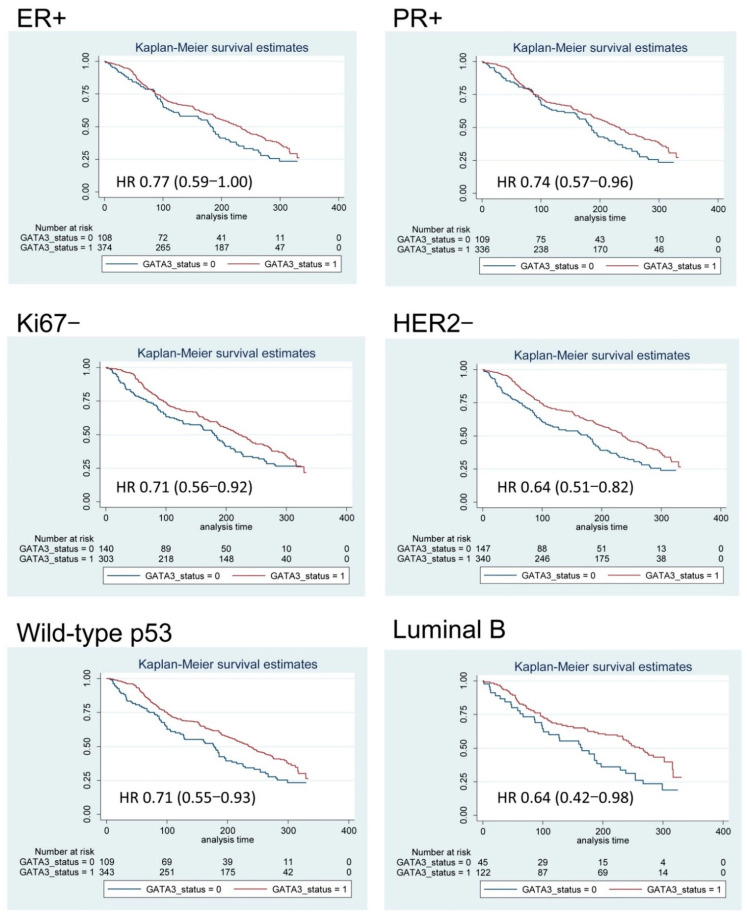
Kaplan–Meier overall survival curves of subgroups of breast cancer patients according to IHC expression of *GATA3*. After adjusting for the patients’ age, *GATA3* IHC positivity was associated with a significantly better overall survival in breast carcinoma patients with positive (+) estrogen receptors (ER) and progesterone receptor (PR), negative (−) HER2 and Ki-67 (i.e., <20%), and p53 wild-type IHC pattern and Luminal B intrinsic subtype. The age-adjusted hazard ratio (HR) for death and the 95% confidence interval (CI) estimated with Cox regression analyses are reported.

In order to explore the prognostic value of *GATA3* positivity in different subgroups, we compared the OS curves from our BC cohort, and stratified according to the main prognostic factors (Table 2, Figure 5, and Supplementary Figure S2). *GATA3* was associated to OS only in women aged 50 years or older. Analysis by grade-, pT-, and stage-specific OS according to *GATA3* IHC showed a better outcome associated with *GATA3* positivity, mainly among patients with a lower grade and lower stage (Table 2 and Supplementary Figure S2). Specifically, *GATA3* positivity was associated with a better prognosis in BC patients older than 50 years, and with an Elston and Ellis grade of 1–2 (HR 0.69, *p* = 0.003), pT1 (HR 0.67, *p* = 0.001), pN0 (HR 0.65, *p* = 0.003), and stage I–II (HR 0.65, *p* < 0.0001). Moreover, *GATA3* positivity maintained the ability to stratify patients with a better prognosis, particularly in BC subgroups with positive ER (HR 0.77, *p* = 0.046), positive PR (HR 0.74, *p* = 0.022), Ki-67 < 20% (HR 0.62, *p* = 0.008), negative HER2 (HR 0.64, *p* < 0.0001), and with p53 wild-type IHC pattern (HR 0.71, *p* = 0.011) (Table 2, Figure 5).

Furthermore, *GATA3* expression was associated with a better OS of BC patients with luminal B intrinsic subtype (median survival 261 vs. 166 months for *GATA3*+ vs. *GATA3*–, HR 0.64, 95% CI: 0.42 to 0.97, *p* = 0.036), but not with the other molecular subtypes (Figure 5). Notably, the opposite trend was observed in patients with positive HER2 (HR 1.06, 95% CI: 0.66 to 1.71, *p* = 0.798), in which *GATA3* positivity was associated with worse OS, but this association was compatible with random fluctuation (Table 2).

According to the log-log plot and predicted versus observed survival curves, *GATA3* was a time-independent variable. As a consequence, we could apply the Cox proportional hazards modeling. After adjusting for all clinico-pathological and IHC variables, stage, *GATA3*, and p53 functioned as independent prognostic factors for BC patients (Table 3).

Differently, based on the log-log plot and predicted vs. observed survival curves, for grade, ER, PR, HER2, Ki-67, and p53, the risks were not proportional, and the Cox model did not correctly predict survival during the whole time period, but just during the first 48 months after surgical treatment. As a consequence, we applied a univariate Cox regression analysis in this limited time period, and found that all covariates had a significant impact on short-term OS (Table S5). Afterward, multivariate analysis on this time period showed that *GATA3* IHC positivity was an independent and favorable predictive factor for improved OS in BC patients, reducing the hazard ratio by 40% (Table 3). In addition, the p53 mutation IHC pattern was an independent risk factor for reduced OS, increasing the hazard ratio two-fold (Table 3). Among the other prognostic factors, only stage and grade remained associated at 48 months. Table S5 shows the association of each biomarker with the 48-month OS adjusted by age and stage.

## 4. Discussion

We found that *GATA3* expression was associated with a 20% reduction in the risk of death during a 28-year follow-up time. Only p53 had a comparable impact on OS, with a 30% higher risk of death. The prognostic value of the two biomarkers was even stronger in the first 48 months of follow-up. No other tumor characteristic showed a stronger association with overall survival.

Moreover, we found that *GATA3* association with OS was also maintained in individual BC subgroups, particularly those at lower risk, such as ER-positive, PR-positive, HER2-negative, with lower proliferation (ki-67 < 20%), p53 wild-type, lower grade, smaller size (pT1–T2), without lymph node metastasis (pN0), at lower stage, and older than 50 years.

*GATA3* is an ER-related gene and, as such, has been associated with improved outcome in BC patients [16,17,22]. However, conclusive data demonstrating *GATA3* as a prognostic factor independent of ER status are still missing [18]. In our study, we found that 68% of BCs expressed *GATA3* by IHC. Previous studies have reported that *GATA3* IHC expression ranged between 31% and 92% in BCs [18,23,24,25]. The apparent discrepancy in *GATA3* expression between these studies may reflect both the methodology used and the different cutoff values assigned to define *GATA3* IHC positivity [26,27]. In our study, we used TMA with relatively large cores, and the positive *GATA3* IHC cutoff was set at 1% (to parallel cutoff percentages of ER and PR), obtaining a high positive rate [1]. Moreover, the assessment of positive IHC can be subjective and operator-dependent; to limit the impact of this variability and to increase score accuracy, we applied an automated scoring method [28].

Consistently with all previous studies, we found an excellent correlation (*p* < 0.0001) between hormone receptor positivity and *GATA3* expression [16,25,29,30]. In fact, in our study, 78% of ER-positive and 76% of PR-positive BCs were also *GATA3* positive, and, vice versa, 93% were also ER-positive and 85% were PR-positive among *GATA3*-positive cases. These results are coherent with the integral role of *GATA3* in the ER signaling pathway revealed by both cell line studies and microarray data analyses [30,31,32]. Moreover, we found a significant association between *GATA3* positivity and HER2 negativity. Our results are consistent with the majority of the previous studies that have investigated this relationship [18,23,33,34], but not with all of them [29,35,36]. Notably, these associations reflect the *GATA3* distribution between BC molecular subtypes.

In addition to usual biological prognostic factors, we evaluated p53 IHC. *TP53* is the most commonly mutated gene in BC, given that *TP53* mutations occur in 30% of BCs [13,14,37]. Importantly, *TP53* mutation has been strongly associated with a worse outcome in BC patients [38,39]. First, we found no association between *GATA3* expression and p53 by IHC percentage, similarly to Hosoda et al. and Jacquemier et al. [36,40]. Conversely, two different studies have identified higher p53 expression in *GATA3*-negative BCs [33,34]. Therefore, we categorized p53 IHC in patterns associated with mutated and wild-type *TP53* status [41,42,43]. In this way, we found a positive association between *GATA3* positivity and p53 wild-type pattern. This novel finding is reasonably expected, since *GATA3* and *TP53* mutations have been shown to be mutually exclusive in BCs [14].

Interestingly, our results in terms of survival associate mutant p53 pattern with an exceedingly worse prognosis at 48 months (HR 2.73) are in agreement with previous studies [38]. Therefore, our results further bolster p53 IHC as a prognostic test in BC tissues.

Looking at clinico-pathological parameters, based on our findings, *GATA3* positivity is inversely associated with the most important clinical prognostic factors of BC outcome, namely tumor size and lymph node metastasis. Most of the previous studies that have examined *GATA3* IHC expression in relation with tumor size and/or the presence of lymph node metastasis did not find any association [23,29,33,44,45,46]; however, four studies analogously reported an inverse association between *GATA3* and tumor size [16,17,18,45], and only one reported low *GATA3* expression associated with lymph node metastasis [16].

Altogether, our findings associate, univocally and clearly, *GATA3* expression to favorable clinical, biological, and pathological features of BC, coherently with and supporting previous observations.

To date, there is still undisguised controversy regarding the ability of *GATA3* to predict the long-term prognosis in BC patients. Specifically, although a high *GATA3* gene expression level has been convincingly associated with a better outcome of BC patients independently of other clinico-pathological features [16,47,48,49], the prognostic value of the *GATA3* protein level by IHC remains inconclusive [16,17,18,35,50].

Among intrinsic molecular subtypes, *GATA3* expression was associated with a better OS in luminal B. It is well-known that different intrinsic molecular subtypes show distinct clinical evolutions. Characteristically, luminal B BC has a poorer outcome compared with luminal A, but similar to HER2-positive and TNBC [51]. Furthermore, luminal B exhibits a delayed aggressive behavior with the highest mortality rate after five to eight years when compared to HER2-positive and TNBC that progress rapidly with the highest mortality during the first two years after the diagnosis [52]. Therefore, our results emphasize the possibility that *GATA3* plays a different prognostic role in various BC intrinsic subtypes. This should be kept in mind for further studies in order to better clarify the additional prognostic information supplied by *GATA3* for therapeutic decision-making, especially in luminal B BCs.

A non-negligible number of studies have investigated the prognostic value of *GATA3* by IHC in continuous series of BCs. The first study by Mehra et al. has analyzed *GATA3* IHC in a cohort of 139 consecutive invasive BCs arranged in TMA blocks, and has found that BC patients with low *GATA3* expression had significantly shorter OS [16]. However, the prognostic value of *GATA3* IHC was not independent of other prognostic variables by a Cox multivariate analysis of OS. Similarly, Voduc et al. could not demonstrate *GATA3* IHC as an independent prognostic predictor in over 3100 consecutive cases of BCs, as well as in the ER-positive subgroup [18]. Analogously, in the same year, Ciocca et al. found a non-significant modest protective effect of *GATA3* positivity in the OS of 166 consecutive BC patients [50]. No association between *GATA3* expression and outcome was found by Albergaria et al. in 249 consecutive BC patients [23]. Thereafter, Yoon et al. could not only confirm the association of *GATA3* IHC positivity with better BC-related survival in a consecutive series of 242 BCs and in the ER-positive subgroup, but also found it in low-grade groups [17]. Two studies in particular have investigated over 200 hormone receptor-positive and HER2-negative BCs, and both found that *GATA3* was significantly associated with a better prognosis in univariate analysis, but not in multivariate analysis [36,45]. Then, McCleskey et al. obtained analogous results by analyzing 62 advanced BC patients [53].

Another study has specifically evaluated the impact of *GATA3* prognostics on 516 BC patients treated with systemic therapy, i.e., chemotherapy and/or tamoxifen [35]. *GATA3* status could not reveal significant differences in OS among these patients, but *GATA3* positivity negatively affected the OS of ER-negative patients (who did not receive tamoxifen), only in the univariate analysis. Finally, two studies investigated the *GATA3* prognostic effect on patients treated with neoadjuvant chemotherapy [54,55]. Both showed that *GATA3* negativity was significantly associated with a more likely complete pathological response [54,55]. Notably, our patients were treated long before the introduction of current BC systemic standard treatment based on the expression of molecular markers and BC molecular intrinsic subtypes, which limits the applicability of our results to present-day patients, but it gives the opportunity to observe its prognostic value in a setting where therapies were given only on the basis of lymph node status, surgical margins, and tumor size.

In summary, altogether, the studies that have investigated *GATA3* as a prognostic factor in BC patients found that, even though *GATA3* is associated with a favorable prognosis, it may not represent an independent prognostic factor, since, in the majority of the studies, *GATA3* could not demonstrate its predictor value in a multivariate analysis [56]. Therefore, *GATA3* has been shown so far as a critical biomarker associated with improved survival, but this association can depend on the association of *GATA3* expression with different variables of good prognosis. Instead, our data showed a strong independent association of *GATA3* positivity with an improved prognosis, particularly in HR-positive BCs and in BCs with a good prognosis. Ours is the second largest study that has analyzed the prognostic impact of *GATA3* IHC, and it is worth noting that results from the two largest studies confirmed an independent association with the prognosis, even in stratified analyses by cancer subtype, that smaller studies could not detect. More generally, our results emphasize the possibility that *GATA3* plays a different prognostic role in various BC subtypes; this should be kept in mind for further studies in order to better clarify the additional prognostic information supplied by *GATA3* for therapeutic decision-making, especially in luminal B BCs. In fact, there is an unmet need to identify patients with luminal A and B cancers who may benefit from adjuvant chemotherapy, particularly among those without early BC [5,57,58,59]. Therefore, given our results, *GATA3* should be explored as a potential biomarker to help define the prognosis of early HR-positive BC, and could demonstrate an immediate, clinically-relevant application in this context and for this specific clinical issue.

## 5. Conclusions

Our study, building on previous observations, provides new evidence of the prognostic value of *GATA3* in BC, inasmuch as it correlates *GATA3* negativity to a worse prognosis, especially in less aggressive BC subgroups. Indeed, *GATA3* IHC could uncover BC patients with worse clinical outcomes in low-risk categories, which would potentially benefit from additional tailored treatment. Our data, therefore, support the possible clinical utility of incorporating *GATA3* IHC analysis into routine practice as an adjunct to standard IHC panel, in order to further risk-stratify BC patients at a remarkably low cost when compared to multigene prognostic tests. Since *GATA3* IHC is currently used in routine diagnostic practice as a surrogate marker for breast and urothelial origins of carcinomas of unknown primary, this should be straightforward, however, standardized methods with a univocal and reproducible cutoff are needed.

## Figures and Tables

**Table 1:** Clinico-pathological characteristics and biological markers of breast cancer patients.

Clinico-Pathological Characteristics	n (%)
Age, total	702
<50 years	166 (23.6)
50–55 years	96 (13.7)
56–70 years	265 (37.7)
>70 years	175 (24.9)
Grade, total	700
1	135 (19.2)
2	427 (60.8)
3	138 (19.7)
Histotype, total	702
No special type	527 (75.1)
Lobular	109 (15.5)
Other special types	66 (9.4)
pT, total	699
T1	450 (64.4)
T2	236 (33.8)
T3	13 (1.8)
pN, total	702
N0	393 (56.0)
N1	184 (26.2)
N2	72 (10.3)
N3	53 (7.5)
Overall survival, failures	
Five-year	119 (17.7)
10-year	237 (33.8)
15-year	291 (41.5)
20-year	375 (53.4)
28-year	432 (61.5)
ER median, range	85.2%, 0–98.9%
ER, total	665
Negative (<1%)	123 (18.5)
Positive (≥1%)	542 (81.5)
PR median, range	57.4%, 0–98.9%
PR, total	663
Negative (<1%)	164 (24.7)
Positive (≥1%)	499 (75.3)
Ki-67 median, range	7.0%, 0–98.2%
Ki-67, total	658
Negative (<20%)	500 (76.0)
Positive (≥20%)	158 (24.0)
HER2, total	676
Negative (0–2+)	555 (82.1)
Positive (3+)	121 (17.9)
p53 median, range	13.0%, 0–98.5%
p53, total	660
Wild-type pattern	503 (76.2)
Mutated pattern	157 (23.8)
*GATA3* score median, range	60, 0–300
GATA3, total	608
Negative (<1%)	195 (32.1)
Positive (≥1%)	413 (67.9)
Molecular intrinsic subtypes, total	646
Luminal A	274 (39.0)
Luminal B	185 (26.4)
Luminal B-HER2+	80 (11.4)
HER2+	37 (5.3)
Triple negative	70 (10.0)

n, number of cases; ER, estrogen receptor; PR, progesterone receptor.

**Table 2:** The prognostic value of *GATA3* positivity stratified by clinico-pathological features and biological prognostic factors. Age-adjusted HRs are estimated through proportional hazard Cox models.

Variable	Stratified Cox Regression Analysis	
	Hazard Ratio	95% Confidence Interval
Age		
<50 years	0.92	0.53–1.60
≥50 years	0.66	0.52–0.83
Histologic Grade		
1–2	0.69	0.54–0.88
3	0.74	0.47–1.16
Histologic Type		
No special type	0.71	0.56–0.91
Lobular	0.69	0.39–1.22
Other	0.35	0.15–0.85
pT		
T1	0.67	0.50–0.90
T2	0.78	0.56–1.08
T3	1.85	0.41–8.41
pN		
N0	0.65	0.48–0.87
N+	0.76	0.56–1.03
Stage	0.69	
I	0.69	0.48–1.00
II	0.66	0.47–0.91
III	0.96	0.61–1.49
ER		
Negative (<1%)	0.55	0.30–1.00
Positive (≥1%)	0.77	0.59–0.99
PR		
Negative (<1%)	0.72	0.47–1.10
Positive (≥1%)	0.74	0.57–0.96
Ki-67		
Negative (<20%)	0.72	0.56–0.92
Positive (≥20%)	0.67	0.44–1.02
HER2		
Negative (0–2+)	0.64	0.51–0.82
Positive (3+)	1.06	0.66–1.71
Molecular intrinsic BC subtypes		
Luminal A	0.78	0.54–1.14
Luminal B	0.64	0.42–0.98
Luminal B-HER2+	1.17	0.62–2.21
HER2+	0.66	0.18–2.36
Triple negative	0.69	0.33–1.46
p53		
Wild-type pattern	0.71	0.55–0.92
Mutated pattern	0.93	0.61–1.41

n, number of cases; ER, estrogen receptor; PR, progesterone receptor.

**Table 3:** Multivariate Cox proportional hazards model for the overall survival.

Multivariate Cox Regression Analysis				
	Entire Follow Up Time *		48-Month Follow-Up	
Variables	Hazard Ratio	95% CI	Hazard Ratio	95% CI
Age	1.06	1.05–1.07	1.03	1.01–1.05
GATA3	0.79	0.63–0.99	0.60	0.37–0.99
p53	1.31	1.02–1.68	2.07	1.24–3.46
Stage				
I	1		1	
II	1.24	0.98–1.57	2.11	1.10–4.05
III	2.55	1.96–3.42	3.84	1.95–7.54
Grade				
1	Not influential		1	
2			2.67	0.81–8.78
3			3.83	1.11–13.24

* Median follow-up, 180 months; CI, confidence interval.

## Data Availability

All data generated or analyzed during this study are included in this published article and its Supplementary Information File.

## Supplementary Materials

The following are available online at https://www.mdpi.com/article/10.3390/diagnostics11040604/s1, Supplementary Material and Methods, Results, Figure S1: distribution by molecular subtypes and *GATA3*, Figure S2: Kaplan–Meier overall survival curves in different breast cancer subgroups, Table S1: primary antibodies and conditions used in this study, Table S2: association between *GATA3* and clinico-pathological characteristics of breast cancer patients, Table S3: correlation between *GATA3* and molecular subtypes, Table S4: Kaplan-Meier survival analysis for the linic-pathological features and biological prognostic factors, Table S5: univariate, age-adjusted, and age and stage-adjusted hazard ratios estimated through proportional hazards Cox regression analysis for the overall survival at 48 months follow-up.
