# Supplementary material for: GATA3 as an Adjunct Prognostic Factor in Breast Cancer Patients with Less Aggressive Disease: A Study with a Review of the Literature
Source: Diagnostics (Basel). 2021 Mar 28;11(4):604. doi: 10.3390/diagnostics11040604 (PMC8066261; doi:10.3390/diagnostics11040604)
Supplement: Supplementary file 1 [file diagnostics-11-00604-s001.pdf]

SUPPLEMENTARY METHODS

Immunohistochemical staining and scoring

Briefly, 4-μm thick formalin-fixed, paraffin-embedded sections were deparaffinized in xylene, rehydrated in graded alcohols and incubated in 3% H_2O_2 in methanol for 10 minutes to block endogenous peroxidase activity. Antigen retrieval was carried out by incubating the slides in Tris-EDTA-citrate buffer (pH 7.8) in a microwave oven, prior to application of primary antibody. Vectastain ABC peroxidase kit (Vector Laboratories, DBA Italia, Segrate, Italy) was applied to reveal antibody binding. The slides were counterstained with hematoxylin for 3 minutes (cat # 790–2208, Ventana Medical Systems/Roche), dehydrated in the graded ethanols and xylene. For each antibody, a positive control slide and a negative control slide, where the primary antibody was replaced with normal serum or isotype-matched antibodies, were included in every staining batch. Endogenous biotin was saturated with a biotin blocking kit (Vector Laboratories).

For nuclear stainings, such as ER, GATA3, Ki-67, p53, and PR, tissue cores were scored as the percentage of positive tumor nuclei above the background using a computer-aided image analyzer (Eureka Interface System, Menarini, Firenze, Italy). For each sample, the number of BC positive nuclei per total number of BC nuclei were counted at high magnification (400x) and reported as the percentage of positive cells. Moreover, the intensity score was also recorded for GATA3. In this way, a GATA3 histological score was obtained, given by the product of the percentage of positive cell nuclei (0%-100%) and the four-tier intensity score (0, 1+, 2+, 3+). Therefore, the final histological score

ranged from 0 to 300 for each core. Except for p53, scoring results were dichotomized into either negative or positive, using the pre-defined threshold values dictated by the 12th *St. Gallen* International Breast Cancer Conference and reported in Table S1 (1). The membrane staining HER2 was considered either negative or positive according to conventional guidelines (2, 3). Differently, p53 was considered to have either a mutated pattern, when it was completely negative (null pattern) or with at least 60% of BC cell nuclei showing intense positivity (missense pattern), or a wild-type pattern when the tumor showed a variable weak-moderate positivity in 1%-59% of cells (4).

Statistical analysis

The features found to be significant in univariate analysis were assessed for the multivariate analysis using enter logistic regression model, to evaluate which features were independent. For multivariate analysis, we compared the *log–log* survival curves and the curves predicted by the Cox model with the observed ones according to the Kaplan-Meier method *to check graphically the proportional hazards assumption for all variables.* The study time endpoint was evaluated starting at total overall survival follow-up (28 years) with a progressive 5-years reduction until 5-years follow-up, then 1-year by 1-year time interval. We used Cox proportional hazards modeling and the likelihood ratio to evaluate survival differences between the different groups (backward parametric statistical Wald method). A setup procedure was used and variables were added to the model if the two-sided significance level was <0.1 in univariate analysis. To control for potential confounding factors, we adjusted HR estimates per age. To evaluate the effect of single variables on patient outcome, the endpoint for overall survival was considered

any death irrespective of cause. Patients without an adverse event were censored at the time of the last follow-up.

SUPPLEMENTARY RESULTS

The median age of the patients at diagnosis was 61 years (range 30-91 years). Among a total of 702 patients included in this study, 513 (73.1%) patients underwent mastectomy or partial mastectomy, whereas the remaining 189 (26.9%) underwent partial resection (lumpectomy, segmentectomy or quadrantectomy). Histologically, 527 (75.1%) carcinomas were no special type, 109 (15.5%) lobular, and 66 (9.4%) were other special types, including 24 tubular, 18 mucinous, 7 papillary, 7 medullary, 6 cribriform, 3 apocrine, and 1 micropapillary.

A total of 424 (60.4%) patients were treated with adjuvant therapy, including 241 (34.3%) patients treated with only tamoxifen-based endocrine therapy, 134 (18.4%) with 6 cycles of cyclophosphamide, methotrexate, and 5-fluorouracil, while 30 (4.3%) with combined chemotherapy and endocrine therapy. Moreover, 186 (26.5%) patients received loco-regional radiotherapy. 154 (21.9%) patients did not receive adjuvant therapy. Overall, 124 (17.7%) patients had incomplete information regarding their medical treatment.

The overall survival rates at 5-years, 10-years, 15-years, 20-years, and 28-years for the 702 cases were 83.0%, 66.2%, 58.5%, 46.6%, and 38.5% respectively, with a average overall survival of 251 months.

A total of 31 TMA blocks were built for this study. Overall, of the 702 BC cores arranged in the TMA, an average of 655 (93.3%) cores per antibody were scorable, whereas on average 47 (7.7%) cores per antibody were unscorable, due to tissue loss, unrepresentative tissue, excessive tissue folding, or non-specific staining per IHC

staining. Altogether, a total of 3930 TMA cores were suitable for IHC evaluation in this study.

Overall, ER and PR were expressed in a higher percentage of BC cells compared with Ki-67 and p53 (85%, 57% vs 7%, 13%, respectively). Moreover, the majority of BC cases were positive (≥1%) for ER (81.5%) and PR (75.3%), negative for HER2 (82.1%), showed a low proliferation index measured with Ki-67 (76.0%) and a p53 wild-type IHC pattern (76.2%). Regarding the molecular subtypes, 646 (92.0%) of 702 could be classified based on immunohistochemistry, where all 4 determinant biological markers ER, PR, HER2, and ki-67 were scored.

REFERENCES

1. Goldhirsch A, Winer EP, Coates AS, Gelber RD, Piccart-Gebhart M, Thurlimann B, et al. Personalizing the treatment of women with early breast cancer: highlights of the St Gallen International Expert Consensus on the Primary Therapy of Early Breast Cancer 2013. Ann Oncol 2013;24(9):2206-23.
2. Wolff AC, Hammond ME, Hicks DG, Dowsett M, McShane LM, Allison KH, et al. Recommendations for human epidermal growth factor receptor 2 testing in breast cancer: American Society of Clinical Oncology/College of American Pathologists clinical practice guideline update. J Clin Oncol 2013;31(31):3997-4013.
3. Querzoli P, Coradini D, Pedriali M, Boracchi P, Ambrogi F, Raimondi E, et al. An immunohistochemically positive E-cadherin status is not always predictive for a good prognosis in human breast cancer. Br J Cancer 2010;103(12):1835-9.
4. Kuhn E, Kurman RJ, Vang R, Sehdev AS, Han G, Soslow R, et al. TP53 mutations in serous tubal intraepithelial carcinoma and concurrent pelvic high-grade serous carcinoma--evidence supporting the clonal relationship of the two lesions. J Pathol 2012;226(3):421-6.

SUPPLEMENTARY TABLES

Table S1: Primary antibodies and conditions used in this study

Antibody	Clone	Vendor	Dilution	Staining location	Threshold value	Stain type
Estrogen receptor alpha	6F11	Ventana Medical Systems Inc.	Prediluted	Nucleus	≥1%	Automated
GATA binding protein 3	HG3-31	Santa Cruz Biotechnology	1:100	Nucleus	≥1%	Manual
HER2/neu	CB11	Cell Marque	Prediluted	Membrane	NA	Automated
Ki-67	Mib1	Biomeda Corp.	1:40	Nucleus	≥20%	Automated
p53	DO7	DBS	Prediluted	Nucleus	0%/≥60%*	Automated
Progesterone receptor	1A6	Ventana Medical Systems Inc.	Prediluted	Nucleus	≥1%	Automated

NA, not applicable.
*intense and diffuse positivity in ≥ 60% of BC cells or complete negativity.

Table S2: Association between GATA3 and clinico-pathological characteristics of breast cancer patients

**Clinico-pathological characteristics**	**n (%)**	**GATA3 negative** (<1%)	**GATA3 positive** (≥1%)	**p-value**
ER, total	591	191	400	
Negative (<1%)	109 (18.5)	83 (76.2)	26 (23.8)	**< 0.0001^**
Positive (≥1%)	482 (81.5)	108 (22.4)	374 (77.6)	
PR, total	588	192	396	
Negative (<1%)	143 (24.3)	83 (58.0)	60 (42.0)	**< 0.0001^**
Positive (≥1%)	445 (75.7)	109 (24.5)	336 (75.5)	
Ki-67, total	587	192	395	
Negative (<20%)	443 (75.5)	140 (31.6)	303 (68.4)	0.3575^
Positive (≥20%)	144 (24.5)	52 (36.1)	92 (63.9)	
HER2, total	596	193	403	
Negative (0-2+)	487 (81.7)	147 (30.2)	340 (69.8)	**0.0175^**
Positive (3+)	109 (18.3)	46 (42.2)	63 (57.8)	
p53, total	586	185	401	
Wild-type pattern	452 (77.1)	109 (24.1)	343 (75.9)	**< 0.0001^**
Mutated pattern	134 (22.9)	76 (56.7)	58 (43.3)	
Age, total	608	195	413	
<50 ys	145 (23.8)	40 (27.6)	105 (72.4)	0.3695#
50-55 ys	74 (12.2)	23 (31.1)	51(68.9)	
> 70 ys	389 (64.0)	102 (26.2)	257 (73.8)	
Grade, total	607	195	412	
1	113 (18.6)	23 (20.4)	90 (79.6)	**< 0.0001#**
2	368 (60.6)	110 (29.9)	258 (70.1)	
3	126 (20.8)	62 (49.2)	64 (50.8)	
Histologic Type, total	608	195	413	
No special type	463 (76.2)	149 (32.2)	314 (67.8)	0.1587#
Lobular	92 (15.1)	24 (26.1)	68 (73.9)	
Other	53 (8.7)	22 (41.5)	31 (58.5)	
pT, total	605	193	412	
T1	383 (64.4)	106 (27.7)	277 (72.3)	**0.0098#**
T2	210 (33.8)	81 (38.6)	129 (62.4)	
T3	12 (1.8)	6 (50.0)	6 (50.0)	
pN, total	608	195	413	
N0	337 (56.0)	106 (31.5)	231 (68.5)	0.8452#
N1	166 (26.2)	52 (31.3)	114 (68.7)	
N2	60 (10.3)	20 (33.3)	40 (66.7)	
N3	45 (7.5)	17 (37.8)	28 (62.2)	
Stage grouping, total	605	193	412	
I	249 (41.2)	64 (25.7)	185 (74.3)	**0.0233#**
II	249 (41.2)	91 (36.5)	158 (63.5)	
III	107 (17.6)	38 (35.5)	69 (64.5)	

n, number of cases; ER, estrogen receptor; PR, progesterone receptor.

Table S3: Correlation between GATA3 and molecular subtypes

**Molecular subtypes**	**n (%)**	**GATA3 negative** (<1%) **n (%)**	**GATA3 positive** (≥1%) **n (%)**	**p-value**
Total	576 (100)	188 (32.6)	388 (67.4)	
Luminal A	241 (41.8)	50 (20.7)	191 (79.3)	**< 0.0001^**
Luminal B	167 (29.0)	45 (26.9)	122 (73.1)	
Luminal B- HER2+	74 (12.9)	20 (27.0)	54 (73.0)	
HER2+	31 (5.4)	25 (80.6)	6 (20.4)	
Triple negative	63 (10.9)	48 (76.2)	15 (23.8)	

^ Chi square test

Table S4: Kaplan-Meier survival analysis for the clinico-pathological features and biological prognostic factors

Variables	Patients n	Deaths n (%)	KM analysis survival Median, mos	HR (95% CI
Age, total	702	432 (61.5)		2.53 (1.95-3.28)
<50 ys	166	65 (39.2)	267	
≥50 ys	536	367 (68.5)	189	
Grade, total	700	431 (61.6)		1.13 (0.89-1.44)
1-2	562	348 (61.9)	213	
3	138	83 (60.2)	207	
Histologic Type, total	699	430 (61.5)		0.86 (0.74-1.00)
No special type	527	327 (62.1)	203	
Lobular	109	71 (65.1)	204	
Other	63	32 (50.8)	297	
pT, total	699	431 (61.7)		1.55 (1.30-1.85)
T1	450	251(55.8)	233	
T2	236	171 (72.5)	153	
T3	13	9 (69.2)	66	
pN, total	702	432 (61.5)		0.64 (0.53-0.78)
N0	393	224 (57.0)	242	
N+	309	208 (67.3)	170	
Stage	699	431 (61.7)		1.56 (1.37-1.77)
I	295	161(54.6)	244	
II	277	170 (61.4)	207	
III	127	100 (78.7)	86	
ER, total	665	414 (62.3)		0.78 (0.60-0.99)
Negative (<1%)	123	75 (61.0)	199	
Positive (≥1%)	542	339 (62.6)	213	
PR, total	663	413 (62.3)		0.82 (0.65-1.03)
Negative (<1%)	164	101 (61.6)	194	
Positive (≥1%)	499	312 (62.5)	214	
Ki-67, total	658	412 (62.6)		1.04 (0.82-1.31)
Negative (<20%)	500	318 (63.6)	207	
Positive (≥20%)	158	94 (59.5)	229	
HER2, total	676	419 (62.0)		1.27 (0.99-1.63)
Negative (0-2+)	555	341 (61.4)	215	
Positive (3+)	121	78 (64.5)	195	
p53, total	660	410 (62.1)		1.40 (1.12-1.75)
Wild-type pattern	503	309 (61.4)	217	
Mutated pattern	157	101 (64.3)	190	
GATA3, total	608	379 (62.3)		0.70 (0.56-0.86)
Negative (<1%)	195	136 (69.7)	181	
Positive (≥1%)	413	243 (58.8)	234	

n, number of cases; HR, hazard ratio; CI, confidence interval; ER, estrogen receptor; PR, progesterone receptor.

Table S5: Univariate, age-adjusted, and age and stage-adjusted hazard ratios estimated through proportional hazards Cox regression analysis for the overall survival at 48 months follow-up.

	Univariate Hazard ratio	95% Confidence Interval	Age-adjusted Hazard ratio	95% Confidence Interval	Age and stage-adjusted Hazard ratio	95% Confidence Interval
Age	1.03	1.01-1.04	-	-	1.02	1.01-1.04
Histologic Grade						
1	1		1		1	
2	3.30	1.32-8.28	3.26	1.30-8.17	2.53	1.00-6.38
3	7.45	2.92-19.06	7.66	3.00-19.58	5.11	1.96-13.27
pT						
1	1		1		-	-
2	2.71	1.77-4.15	2.64	1.73-4.06		
3	3.09	0.95-10.04	3.41	1.05-11.06		
pN						
0	1		1		-	-
1	2.37	1.54-3.65	2.41	1.57-3.72		
Stage						
I	1		1		-	-
II	2.49	1.41-4.41	2.74	1.39-4.37		
III	5.20	2.91-9.31	5.08	2.84-9.11		
ER	0.43	0.27-0.67	0.41	0.26-0.65	0.44	0.27-0.69
PR	0.46	0.29-0.71	0.45	0.29-0.70	0.48	0.31-0.74
Ki-67	1.44	0.90-2.21	1.60	0.99-2.56	1.61	1.00-2.58
HER2	1.76	1.09-2.84	1.86	1.15-3.00	1.52	0.94-2.48
p53	3.29	2.14-5.06	3.49	2.26-5.37	3.14	2.03-4.84
GATA3	0.38	0.25-0.60	0.40	0.25-0.62	0.43	0.27-0.68

ER, estrogen receptor; PR, progesterone receptor.

SUPPLEMENTARY FIGURES

Figure S1

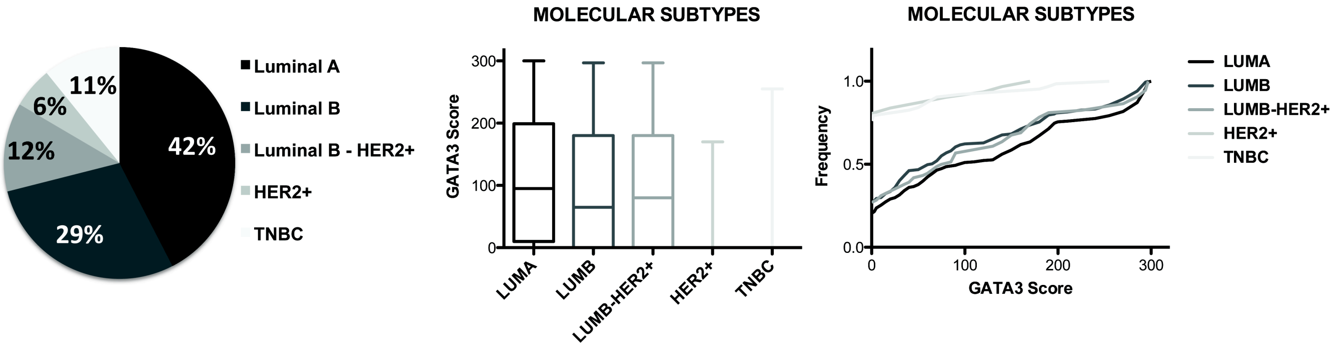

Figure S1. Pie chart representing the distribution by and molecular subtypes of our series of breast carcinomas (left panel). Box-plot (middle panel) and cumulative relative frequency charts (right panel) show that GATA3 histological score was significantly higher in luminal intrinsic BC subtypes ($p<0.0001$) when compared to HER2-positive (+) and triple-negative (TNBC) subtypes.

Figure S2

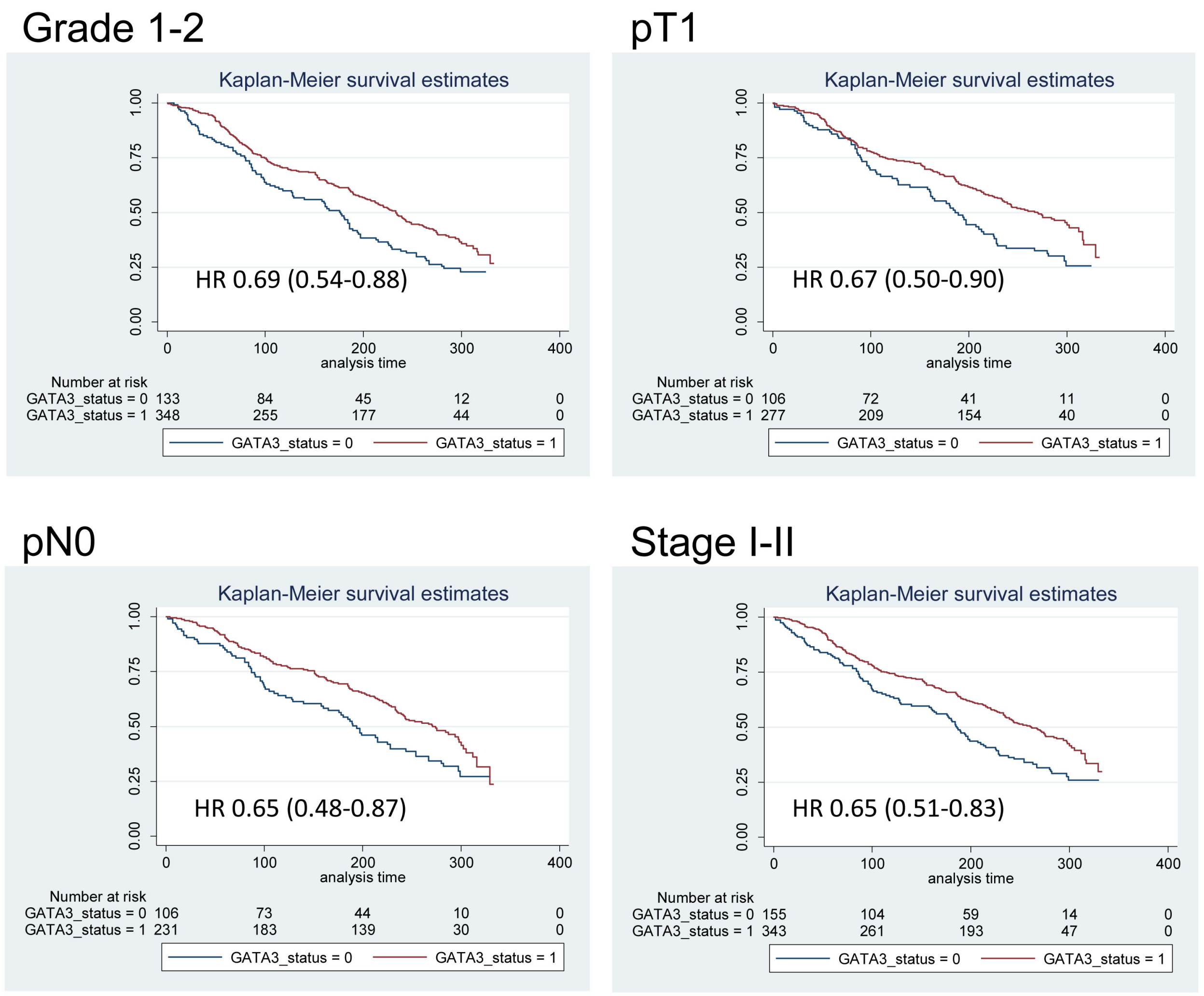

Figure S2. Kaplan–Meier overall survival curves of subgroups of breast cancer patients according to IHC expression of GATA3. After adjusting for the patients' age, GATA3 IHC positivity is associated with a significant better overall survival in breast carcinoma patients with histological grade 1 and 2, pT1-T2, pN0 and Stage I and II. The age-adjusted hazard ratio (HR) for death and the 95% confidence interval (CI) estimated with Cox regression analyses are reported.
